# Olfactory detection of prey by the termite-raiding ant *Pachycondyla analis*

**DOI:** 10.1093/jis/14.1.53

**Published:** 2014-01-01

**Authors:** Abdullahi Ahmed Yusuf, Robin M. Crewe, Christian W. W. Pirk

**Affiliations:** 1 Social Insects Research Group, Department of Zoology and Entomology, University of Pretoria, Private Bag X20, Hatfield 0028 Pretoria, Republic of South Africa; 2 *icipe* , P. O. Box 30772-00100 GPO Nairobi, Kenya

**Keywords:** Matabele ants, *Megaponera foetens*, odors, olfactory cues, Ponerine ant, termite gallery soil

## Abstract

The African termiteraiding ant *Pachycondyla analis* Latreille (Hymenoptera: Formicidae) organizes group raids on termites of the subfamily Macrotermitinae. Termites and ants occupy and share similar habitats, resulting in a co-evolutionary arms race between termites as prey and ants as predators. The present study explored whether *P. analis* uses semio- chemical signaling cues to detect potential termite prey prior to and during raids. Ants’ responses to odors emitted from termites alone, termite gallery soil, and termites inside their galleries were tested using Y-tube olfactometer assays. The results showed that *P. analis* detected odors of termites and those of their galleries, and odors from termites inside their galleries were more attractive to both minor and major ant workers than odors from termites alone. The composition of these odor sources was identified using gas chromatography-mass spectrometry analysis. While the odors from termite gallery soils were compositionally richer (containing 13 compounds rather than nine from termites alone), those from the termites alone were quantitatively richer, releasing about six times more odors than gallery soil. Most of the compounds in the odor profiles were identified as hydrocarbons. Naphthalene, previously identified as an insect repellent, was also identified as a component of the odors from the gallery soil. These results demonstrate that odors play an important role in prey detection by *P. analis* .

## Introduction

Ants are the greatest predators of termites worldwide ( Hölldobler and Wilson 1990 ). Ants and termites share the same habitats and are abundant in terms of biomass and density ( Fujiwara-Tsujii et al. 2006 ). During their 100 million years of coexistence, some species of ants and termites have engaged in a coevolutionary arms race, with ants developing several predatory tactics and termites defending themselves ( Deligne et al. 1981 ; Mill 1983 ; Hölldobler and Wilson 1990 ). Despite these counteracting strategies from termites, most ant-termite interactions are antagonistic, and the well-armed ants tend to win battles against the soft-bodied termites. Among ants there are specialist as well as opportunistic predators of termites. Ants that prey opportunistically on termites belong to two of the largest genera, *Pheidole* spp. and *Camponotus* spp. ( Hölldobler and Wilson 1990 ). The most specialized predatory ant species are concentrated in the subfamilies Ponerinae and Myrmicinae ( Lévieux 1966 ; Maschwitz and Mühlenberg 1975 ; Longhurst et al. 1978 , 1979; Maschwitz and Schönegge 1983 ; Lepage 1984 ; Corbara and Dejean 2000 ).

A large amount of energy and time is used by virtually all animals in searching for food, regardless of social organization ( Bell 1991 ). For a colony of ants, foraging is energetically expensive but ultimately pays off with an increase in the probability of retrieving more food items ( Lighton et al. 1987 ). In ants, scouts have the ability to learn and recognize prey characteristics such as spatial distribution and availability ( Hölldobler and Wilson 1990 ; Schatz et al. 1999 ) or specific odors or kairomones (Durou et al. 2000). This has potential to increase ants’ foraging efficiency if scouts are able to recruit nest mates to the sites of prey. Since termite availability is determined by their complex spatial and temporal presence (foraging and nesting habits), their exploitation as prey requires some synchronization on behalf of predators, as observed in the raiding behavior of the African termiteraiding ant, *Pachycondyla analis* Latreille (Hymenoptera: Formicidae) ( Yusuf et al. 2014 ).

*P. analis* is a specialized predator of termites that is widely distributed in sub-Saharan Africa ( Lévieux 1966 ). This ant species, commonly referred to as Matabele ants, organizes group raids on termite species in the subfamily Macrotermitinae ( Longhurst et al. 1978 ). These raids are initiated when a scout ant detects a potential food source and then recruits nest mates using trail pheromones ( Longhurst et al. 1979 ). Upon arrival at the food source, the ants spread out, break open the termite galleries, and invade them to seek the termites. *Pachycondyla analis* captures termites by stinging them, which results in paralysis. The ants then carry the paralyzed termites out of the gallery to a place near the gallery entrance and return to the gallery to continue hunting. After gathering enough termites, they stop hunting, re-group in columns, and start the return journey back to their nest ( Longhurst et al. 1978 ). A major worker can carry up to seven termites between its mandibles, while a minor can carry up to three termites (Video 1, available online at http://insectscience.org/15.53/Yusufvid1.wmv ). Some ants lead the columns of nest mates on the return journey to the nest and do not carry any termites ( Longhurst et al. 1978 ). The raids last between 4–50 min depending on the foraging distance and the termite species being raided ( Yusuf 2010 ).

The cues involved in prey detection by *P. analis* scouts have not been well-studied. Previous studies by Longhurst and Howse (1977 , 1978) reported that *P. analis* scouts either use kairomones or mechanical cues of termite origin to detect potential termite prey. However, New (1991) was of the opinion that *P. analis* scouts detect termites using termite pheromones, which serve as kairomones for *P. analis* . The suggestion by Longhurst and Howse (1977) that the ants are using “extractable chemicals from termites incorporated into soil sheets” has not been supported by experimental evidence, which shows that the cues can be detected by *P. analis* while foraging for termites.

The present study was designed to test whether pheromones emitted by the termites act as kairomones for the ants to seek and capture their prey. Responses of worker ants to the odors of termites, termite galleries (“soil sheets”), and termites inside their galleries were used to identify potential sources of kairomones. The composition of the chemical components from these odor sources was identified using gas chromatography-mass spectrometry (GC-MS) analysis.

## Materials and Methods

### Study insects

Six colonies of *P. analis* with all representative individuals (queen, workers, males, brood, and eggs) were excavated from nests at Mpala Research Centre (0° 17' N, 37° 52' E) in Central Kenya, 250 km north of Nairobi. The ant colonies were kept in artificial nest boxes (20 × 20 × 20 cm) made of aluminum that were connected to foraging arenas (1.5 × 1.0 m) made of Perspex ( www.perspex.com ). The nests were maintained at 25 ± 1°C, 50– 60% relative humidity, and a 12:12 L:D photoperiod ( Yusuf et al. 2013 ). Ants were fed live termites (mainly from the subfamily Macrotermitinae) collected twice daily around the *icipe* (African Insect Science for Food and Health) campus of Duduville in Nairobi, Kenya. Termites ( *Odontotermes* sp.) and gallery soils used for bioassays were obtained from termite foraging galleries in and around the Duduville campus.

### Bioassays

The olfactory responses of major and minor *P. analis* workers to odors were tested in a ytube olfactometer. The odor source consisted of: (a) 40 workers and 10 soldiers (termites only), (b) 250 g gallery soil (termite gallery soil only), (c) a combination of (a) and (b), and (d) a choice between (a) and (c), but this time increasing the number of termites in (a) to 100 (80 workers and 20 soldiers). In order to simulate ant foraging and raiding behavior as observed in the field, all bioassays were carried out in the mornings (07:00–10:00) and evenings (16:00–17:30) over a number of days using ants from six different colonies.

### Y-tube olfactometer

The olfactometer consisted of a glass Y-tube (base 7.5 cm long; Y-arms each 7.5 cm long; internal tube 10 mm outer diameter). The Y-tube apparatus was modified after the design of Carroll et al. (2006) . The two arms and base tube of the olfactometer were connected to Teflon tubes of similar size that were attached directly to the odor and vacuum sources. A mesh screen was placed at each end of the olfactometer to prevent test ants from getting out of the test arena into the Teflon tubing. Odor sources were placed in 200 mL glass chambers with screw tops containing inlets for incoming air and outlets for odors to exit into the Y-tube. Charcoal- purified air was passed into the odor chambers at a flow rate of 250 mL/min. One of the Y- arms was connected to an odor source while the other was connected to an empty jar with only clean air (blank) passing through, except in the case of (d) above. The odors were extracted through the base arm at 500 mL/min by a vacuum pump to ensure a steady flow and to prevent odors from building up in the Y-tube.

Test ants were introduced individually by disconnecting the Y-tube at its base and allowing the ant to walk into the olfactometer. Subsequently, the tube was reconnected to reestablish the airflow from the odor sources through the arms and out at the base towards the vacuum pump. Each ant was allowed to settle down for 5 min, after which its behavior was monitored. A choice was recorded when an ant stayed for at least 1 min in an arm, or when it frequently visited an arm. No choice was recorded when the ant remained in the base arm for more than 5 min. Each test was terminated after 10 min from the introduction of the ant into the Y-tube. Sixty ants were used for each treatment (30 minor and 30 major workers). To avoid positional bias, odor chambers were switched after every replicate. A clean Y-tube was used for each ant test in order to avoid carryover of odors. Parts between the Y-tube, vacuum, and odor sources were changed or cleaned with soapy water and rinsed with Dichloromethane and acetone after each bioassay to remove traces of odors or contaminants. Glassware was cleaned with Teepol ( www.teepol.co.uk ) laboratory detergent, rinsed with acetone, and dried for five hours at 160°C in an oven. Teflon parts were rinsed with acetone and water to remove odors and then flushed with a stream of nitrogen to dry.

### Extraction of compounds and chemical analyses

Approximately 2 g of termite gallery soil was weighed into a clean 2 mL glass vial, and to this 1 mL of *n* -pentane was added. The sample was vortexed for about 10 min, and then extracted for 2 hr at room temperature, after which the supernatant was filtered through solvent-cleaned glass wool and concentrated under charcoal-purified nitrogen to about 100 µL. If samples were not analyzed immediately, they were stored in the freezer at -20°C until used.

Ten termites that were previously killed on ice were extracted in 1 mL of *n-* pentane kept on ice for 2 hr. After extraction, the extracts were filtered through glass wool and the filtrate concentrated under nitrogen to 100 µL. Extracts were either analyzed immediately or stored at -20°C until used.

### GC/GC-MS analyses

GC analysis was carried out on an HP 5890 Series II gas chromatograph (Hewlett Packard, www.hp.com ) equipped with a flame ionization detector and an HP-5 column (30 m × 0.25 mm ID × 0.25 µm film thickness). Nitrogen was used as a carrier gas, with a column pressure of 46 psi and injection temperature of 250°C. One µ L of sample was injected in the splitless mode, with the oven temperature programmed at 60°C for 5 min and increased at 10°C/min to 250°C, and held at this temperature for 13 min. GC-MS analysis was carried on an Agilent Technologies 7890A gas chromatograph ( www.agilent.com ) equipped with a capillary column HP-5 MS (30 m × 0.25mm ID × 0.25 µm film thickness) (Hewlett Packard) and coupled to a 5795C mass spectrometer. One µ L of each sample was injected in the splitless mode, and helium was used as the carrier gas at 1.0 mL min-1. The oven was programmed at 35°C for 5 min, increased to 250°C at 10°C min-1, and then held at this temperature for 15 min. The analysis was carried out at 70 eV in the electron impact ionization mode. Identities of the compounds were confirmed using commercially- available synthetics (where possible) and tentatively based on comparison with published mass spectra and retention indices.

### Statistical analyses

Data analysis was carried out using SAS version 9.2 (SAS Institute, www.sas.com ). Data obtained using the Y-tube olfactometer assays were analyzed using a chisquare test to test whether odors were more attractive to ants than the control (blank). Ants that did not make a choice were not included in the analysis. Because the olfactometer assays were performed under the same conditions, individual assays were pooled in order to evaluate the differences in attractiveness of the three odor sources for major and minor workers. A logistic regression model was fitted to the data using PROC GENMOD.

## Results

### Bioassays

**Figure 1.**
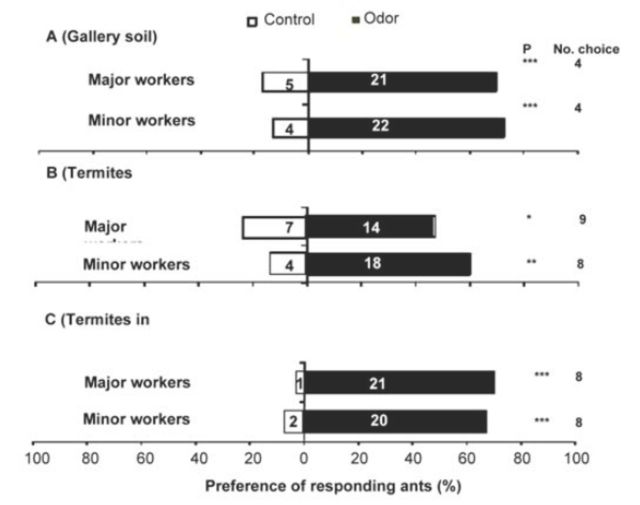
Preferences of *Pachycondyla analis* major and minor workers for odors from: A. *Odontotermes sp* . gallery soil; B. *Odontotermes* sp. workers and soldiers; and C. *Odontotermes* sp. and gallery soil when presented alongside clean air. Black bars represent response to odors, white bars represent response to the control. Numbers within bars refer to the number of ants making a choice, and numbers outside bars refer to ants that made no choice (N *=* 30 each for major and minor workers in each test, ** = significant at *P <* 0.05 and *** = significant at *P* < 0.001). High quality figures are available online.

In general, significantly more ants (65%) responded to the treatment odors than to the control (clean air). Responses of both major and minor workers to termite gallery soil were significantly higher than the control ( Figure 1A ). Twenty-one majors, representing 81% of all major workers, responded to the gallery soil odor, as compared to the 19% that responded to the control (χ2 = 11.56, *P <* 0.001, n = 26). In the case of minors, 85% chose the gallery soil and 15% chose the control (χ2 = 12.46, *P <* 0.001, n = 26). Minors (82% vs.18% control; χ2 = 8.90, *P <* 0.01, n = 22) were more responsive to termite odors than majors(67% vs. 33% control; χ2 = 4.33, *P <* 0.05, n = 21) ( Figure 1B ). Both majors (95% vs. 5% control χ2 = 18.18, *P <* 0.001, n = 22) and minors (91% vs. 9% control; χ 2 = 14.72, *P <* 0.001, n = 22) were highly attracted to the odors from termite galleries ( Figure 1C ).

**Figure 2.**
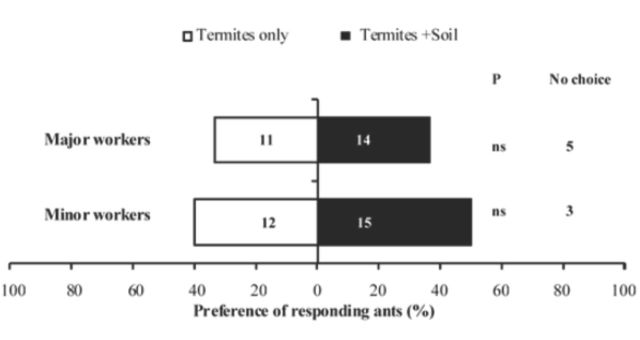
Preferences of *Pachycondyla analis* major and minor workers to odors from *Odontotermes* sp. (workers and soldiers vs. odors from *Odontotermes* sp. in gallery soil. Black bars represent response to odors from termites in gallery soil, white bars represent response to odors from termites only. Numbers within bars refer to the number of ants making a choice, and numbers outside bars refer to ants that made no choice. N *=* 30 each for major and minor workers in each treatment, ns = not statistically significant at α = 0.05. High quality figures are available online.

Given a choice between odors from termites only or termites in the gallery soil, the difference in the response of both major and minor workers to these odors were not statistically significant (majors: χ2 = 0.33, *P =* 0.56, n = 22; minors: χ2 = 11.56, *P =* 0.67, n = 27) ( Figure 2 ). When the responses of both major and minor workers were pooled for all the odors tested, the response of workers to the odors from the gallery soil and those from the gallery with termites inside was not significantly different ( *P* = 0.54). However, responses of the ants to odors from termites and to the combined odors from termites and gallery were significantly different ( *P* = 0.04), with no differences between the responses of major and minor workers ( *P =* 0.84).

In choice tests with termite odors only and those from termites in galleries, both worker ant castes responded more to the odors from termites in galleries than to odors from termites alone ( Figure 2 ). Although the ants did not show any strong preference for termites in galleries over termites alone, the presence of termites in galleries could be used by scout ants as an olfactory cue for choosing galleries to attack. For a scout to make a decision to label a gallery as a potential food source, it has to detect the presence of termite prey inside the galleries by the use of either chemical or mechanical cues of termite origin.

### Identification of chemicals

**Figure 3.**
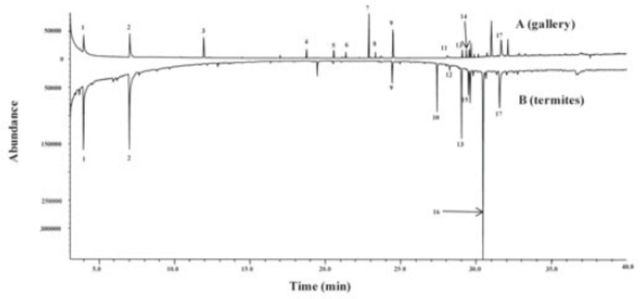
GC-MS trace of chemical compounds extracted from: A. ~2 g of *Odontotermes* sp. gallery soil, and B. 10 *Odontotermes* sp. workers. Labeled peaks are: 1) n *-* heptane; 2) n *-* octane; 3) α-Phallendrene; 4) naphthalene; 5) Butanoic acid-tridecyl-ester; 6) 2-Napthalenemethanol; 7) Methyl-carbinol; 8) n *-* heptadecane; 9) n *-* eicosane; 10) n *-* tetracosane; 11) n *-* pentacosane; 12) hexylpentadecane; 13) 13-undecylpentacosane; 14) n *-* octacosane; 15) 1-Nonadecene; 16) Oxalic acid, hexyl pentadecyl ester; 17) squalene. High quality figures are available online.

Using GC-MS, 17 components were identified from the odors of the termite gallery soil and the termites only ( Figure 3 ). These components were mainly hydrocarbons and esters. The odors from the termite gallery were compositionally richer, with 13 components compared to nine in the odor profile of termites ( Figure 3 ). A combination of the two sets of odors seems to have the potential to enhance the response of the scouts ( Figures 1C and 2 ).

## Discussion

The results from the Y-tube olfactometer bioassays showed that workers of *P. analis* were attracted by olfactory cues associated with both termites and termite galleries, which suggests that they use both to locate prey. The combined odors from termites in their galleries were most attractive to both major and minor ant workers, and it appeared that major ant workers were more sensitive to detecting these odors than minors. This difference in sensitivity may be associated with the fact that major workers are frequently engaged in scouting for food and possibly have an enhanced ability to detect these potential food sources for the colony. The specialization of major workers in locating potential food sources accurately was observed in the field during raids, of which only five (out of 330) observed raids were unsuccessful ( Yusuf 2014 ).

Longhurst and Howse (1978) performed behavioral assays with scout ants (major workers) in the field and found that the ants responded most to dry soil sheeting containing live termites; they speculated that the ants were responding to mechanical cues of the termites drumming their heads on the soil sheets. However, in bioassays involving extracts of a variety of materials, Longhurst and Howse (1978) discovered that ants responded differently to these extracts, indicating that they can respond both to olfactory and mechanical cues. In the present study, field observations showed no raids by ants on fresh termite soil, and as such this type of soil was not included in the study. Furthermore, in preliminary assays, ants did not discriminate bebetween odors from wet soil sheeting and the clean air. The results revealed that *P. analis* workers use olfactory cues to locate termite prey accurately in the absence of visual or mechanical cues, which adds a new dimension to *P. analis* host location.

Comparatively, minor workers responded more to odors from termites only and termite galleries than major workers did. These slight differences were not apparent when odors of the termites in the galleries were offered to different worker groups against a blank. The differences in the responses may be associated with the type of tasks minor workers undertake most, especially during raids. During raids, the smaller body size of minor workers allows them to enter deep inside the termite galleries to seek, paralyze, and carry prey from the galleries. A similar sensitivity to olfactory cues of chemical origin by minor workers was observed in assays that were carried out on volatile cues from conspecific *P. analis* workers (Yusuf et al. unpublished data).

Although this is the first demonstration that *P. analis* use olfactory cues for detecting prey, a previous study had reported a similar detection mechanism in the Myrmicinae ant, *Crematogaster scutellaris* , which used olfactory cues to detect its fig wasp prey ( Schatz et al. 2003 ). The use of allomones in detecting termites has also been described for the larvae of *Lomamyia latipennis* ( Johnson and Hagen 1981 ).

In the present study, chemical profiles of gallery soils and those of *Odontotermes* sp. (termites) were found to be different, with the exception of five hydrocarbons ( *n-* heptane, *n-* octane, *n-* eicosane, *n-* pentacosane, and squalene) common to the profiles of these two antattractive sources ( Figure 3 ). The odors from the gallery soil were qualitatively richer than those from the termites. However, about sixfold more odors were released by the termites ( Figure 3 ). This is the first time the potential chemical cues from both termites and their galleries have been identified in relation to the raiding behavior of *P. analis* . The presence of naphthalene and its derivative 2-naphthalenemethanol in the odors of the galleries, which are known insect repellents, had previously been reported for subterranean termites (McLaughin 2004). Termites use naphthalene and related compounds as a repellent against other insects, especially ant predators. Naphthalene and a naphthalene derivative of plant origin (2-acetonaphthone) have also been reported as repellents for termites ( Henderson et al. 2007 ). The chemicals in the termite soil galleries are believed to come from intestinal secretions of workers, who mix them with soil particles and their saliva ( Briunsma and Leuthold 1977 ). These chemical components of termite origin embedded in the galleries could be used by *P. analis* scouts as an indicator of the presence of termites before detecting other cues associated with the termites themselves, such as mechanical cues like vibrations.

The ability of *P. analis* to be attracted to odors from termite galleries that include the presence of known ant repellents shows that this ant could have evolved to use naphthalene and its derivatives of termite origin as possible kairomones in addition to other components from the cues.

In conclusion, this study investigated the roles of olfactory cues in the detection of termite prey by the termite-specific ant *P. analis* . The results suggest that ant scouts initially detect chemical cues (which act as possible kairomones) from termite galleries and then use a combination of cues of termite origin (which could include mechanical cues) and those of galleries to identify a potential food source and initiate a raid. Both major and minor workers of *P. analis* detect these chemical cues. However, the identity of the behaviorally-active components contributing to detection of the termite food source, either separately or in combination, needs to be established in future research.
